# Allergic sensitization in Canadian chronic rhinosinusitis patients

**DOI:** 10.1186/1710-1492-10-15

**Published:** 2014-03-25

**Authors:** Brett J Green, Donald H Beezhold, Zane Gallinger, Carly S Barron, Rochelle Melvin, Toni A Bledsoe, Michael L Kashon, Gordon L Sussman

**Affiliations:** 1 Allergy and Clinical Immunology Branch, Health Effects Laboratory Division, National Institute for Occupational Safety and Health, Centers for Disease Control and Prevention, 1095 Willowdale Road, M/S L-4020, 26505 Morgantown, WV, USA; 2 Gordon Sussman Clinical Research Inc., Allergy and Clinical Immunology, Toronto, Ontario, Canada; 3 Biostatics and Epidemiology Branch, Health Effects Laboratory Division, National Institute for Occupational Safety and Health, Centers for Disease Control and Prevention, Morgantown, West Virginia, USA

**Keywords:** Allergic rhinitis, Allergic sensitization, Chronic rhinosinusitis, Perennial allergens

## Abstract

**Background:**

Chronic rhinosinusitis (CRS) is a societal burden and cause of morbidity in Canada; however, the prevalence of allergic sensitization in Canadian CRS patients has remained poorly characterized.

**Objective:**

In this study, we used skin prick test (SPT) and specific immunoglobulin E (sIgE) and G (sIgG) titers to regionally relevant allergen sources in order to determine whether allergic sensitization is more prevalent in CRS patients compared to chronic idiopathic urticaria (CIU) control patients.

**Methods:**

One hundred and fifty eight subjects (19–70 years of age) were recruited into the study. 101 subjects had a confirmed diagnostic history of CRS and 57 subjects with a clinical diagnosis of CIU were recruited as controls. Enrolled subjects underwent SPT to a panel of perennial and seasonal allergens and sIgE titers were quantified to selected environmental allergen mixes (grass, mold, and tree species) using Phadia ImmunoCAP. sIgG was additionally quantified to *Alternaria alternata*, *Aspergillus versicolor, Cladosporium herbarum*, and *Stachybotrys atra*. Differences between CRS and control CIU patient SPT and serological data were examined by chi-squared analysis and analysis of variance.

**Results:**

Reactivity to at least one SPT extract occurred in 73% of CRS patients. Positive SPT reactivity to *A. alternata* (odds ratio (OR): 4.34, 95% confidence interval: 1.57, 12.02), cat (OR: 3.23, 95% CI: 1.16, 9.02), and ragweed (OR: 2.31, 95% CI: 1.02, 5.19) extracts were more prevalent in patients with CRS (p < 0.05). Although dust mite and timothy grass sensitization approached statistical significance in the chi-squared analysis of SPT data, other common perennial and seasonal allergens were not associated with CRS. No statistically significant differences were observed between mean sIgE and sIgG titers in CRS and control patients.

**Conclusions:**

This study supports previous data that suggests *A. alternata* sensitization is associated with CRS; however, these findings additionally highlight the contribution of other regionally important allergens including cat and ragweed.

## Background

Chronic rhinosinusitis (CRS) is characterized by inflammation of the nasal mucosa and one or more paranasal sinuses for temporal intervals extending 8–12 weeks [1-3]. In Canada, CRS is an increasing public health burden and accounted for approximately 3 million dispensed prescriptions in 2006 [4,5]. Several Canadian epidemiological surveys have reported the prevalence of CRS to affect approximately 5% of the general population [5,6]. In those studies, the prevalence of CRS was higher in women compared to men and eastern provinces had the highest prevalence of CRS [5,6]. In Canada, like the United States, CRS causes significant morbidity and reduced workplace productivity [2,5,7]. In the National Population Health Survey, Canadian survey participants that were identified with CRS had a self-perceived health status comparable to participants with other chronic conditions such as cancer, arthritis, asthma and inflammatory bowel disease [5]. A reduction in the mental health of survey participants with CRS was also reported and associated with a higher prevalence of depression, anti-depressant use, and number of visits to mental health professionals [5]. These results highlight the emerging societal burden of CRS; however, fewer studies have characterized the allergic sensitizations associated with cases of CRS in Canada.

Studies of CRS etiology have shown that a progressive accumulation of mucus can result in remodeling and obstruction of the sinus ostia, bone erosion, facial pain, swelling, and can co-occur with or without polyp formation [1]. Although CRS may have an infectious or non-infectious basis [1,8], allergic sensitization has been suggested in several studies as an important contributing factor to the underlying pathology [3,6,9-11]. Total IgE levels have been shown to correlate with the extent of CRS disease on sinus CT [12]. Inflammation, swelling and decreased mucociliary clearance associated with allergic rhinitis and eosinophilic inflammation have been postulated to enhance paranasal sinus ostia destruction [6,9,10]. This pathology may increase the risk of CRS development in sensitized patients but to date, the role of allergens and allergic sensitization remains uncharacterized and requires further clinical evaluation.

The coexistence of allergic rhinitis and CRS has been previously reported in the literature [9,10,13,14] including in a Canadian study [6]. The prevalence of allergic sensitization has been reported to be as high as 84% in patients with CRS [9,13] and a major subset of this patient group can be sensitized to more than one allergen [9]. Perennial allergen sensitization is more prevalent in CRS patients [14] with dust mite and mold allergens among the most frequently reported sensitizing sources [9,14]. The role of fungi in CRS has been the focus of many research studies as these microbial contaminants are commonly recovered from eosinophilic/allergic mucin. The association with fungi has been controversial but is sometimes described as mediating host immune reactions leading to the development of CRS [8,15]. Despite these experimental findings, the contribution of fungi and other perennial allergens to CRS in Canada has remained poorly characterized.

In this study, we document the prevalence of allergic sensitization to a selection of regionally prevalent perennial and seasonal allergen sources in a cohort of Canadian CRS patients. In addition, we compare the prevalence of allergic sensitization to a wider panel of fungi implicated in CRS. The identification of allergic sensitization to regionally prevalent allergens may be an additional diagnostic marker of CRS in clinical populations.

## Materials and methods

### Human subjects

One hundred and fifty eight study participants from the Toronto metropolitan area (19–70 years of age) were recruited into the study. One hundred and one subjects were diagnosed with a history of CRS and 57 subjects were recruited as controls with chronic spontaneous urticaria (CIU). These study participant numbers were not based on a sample size calculation. CRS was physician diagnosed by physical exam based on the following symptomology: sinus congestion or fullness; facial pain, pressure or fullness; nasal obstruction or blockage; purulent anterior or posterior nasal drainage; and decrease sense of smell [16]. All CRS patients had endoscopy and CT scans confirming diagnosis of CRS. CIU is generally considered a non-IgE mediated disease and physician diagnosed patients were recruited to the control population as a way to control for confounding variables. When possible, consecutive patients presenting to the clinic were recruited to this study.

Enrolled subjects underwent skin prick testing (SPT) and were asked to provide a blood sample by venipuncture (20 mL) for serological analysis of specific immunoglobulin E (sIgE) and G (sIgG). Human serum was separated *in vitro* from the collected blood samples and then transferred to the National Institute for Occupational Safety and Health (NIOSH) and stored at −80°C until serological analysis. This study was approved by the NIOSH Human Subject Review Board (05-HELD-04XP) and the Ontario Institutional Review Board Services. Informed consent was obtained from all participants.

### Skin prick testing

The panel of SPT extracts comprised a variety of perennial (cat, dog, dust mite, cockroach and mouse), grass/weed (june, orchard, sweet timothy, cocklebur and ragweed), tree (ash, black birch, elm, maple, oak, pine, poplar and willow) and fungal allergens (*Alternaria alternata*, *Aspergillus flavus*, *Cephalosporium*, *Cephalothecium*, *Fusarium*, *Helminthosporium*, *Hormodendrum*, *Monilia sitophila*, *Mucor*, *Penicillium*, *Phoma*, and *Rhizopus)* from ALK (Hørsholm, Denmark). SPTs were performed by placing a drop of test solution on the skin and pricking the epidermis beneath the drop with a disposable plastic lancet (J.N. Eberle GmbH Hochfeldrasse 6–8 D-86830 Schwabmuenchen, Germany). SPT sites were wiped clean and after fifteen minutes, the wheal and flare reactions were outlined and the diameters measured. Histamine (10 mg/mL) was used as a positive control and diluent (HSA in 50% glycerol) was used as a negative control. A positive reaction was characterized as 3 mm or greater than that of the negative control.

### Serological analysis

Serological quantification of sIgE and sIgG titers was conducted by fluoroenzyme immunoassay using a Phadia ImmunoCAP 100 (Phadia AB, Uppsala, Sweden). sIgE was quantified to selected environmental allergens using a sIgE multiple allergen mix to grass (gx1), molds (mx2), and trees (tx1). The grass mix (gx1) was comprised of *Dactylis glomerata* (g3), *Festuca elatior* (g4), *Lolium perenne* (g5), *Phleum pratense* (g6), and *Poa pratense* (g8). The mold mix (mx2) was comprised of *Penicillium chrysogenum* (m1), *Cladosporium herbarum* (m2), *Aspergillus fumigatus* (m3), *Candida albicans* (m5), *A. alternata* (m6), and *Setomelanomma rostrata* (m8). The tree pollen mix (tx1) was comprised of *Acer negundo* (t1), *Betula verrucosa* (t3), *Quercus alba* (t7), *Ulmus americana* (t8), and *Juglans californica* (t10). sIgE was considered detectable when 65 allergen specific units (kUA/L) were exceeded. sIgG titers were also measured using ImmunoCAP for a 4-mold panel: *C. herbarum* (Gm2), *A. alternata* (Gm6), *Stachybotrys atra* (RGm24), and *Aspergillus versicolor* (RGm25). sIgG <0.02 mg/L was considered undetectable as previously described [17].

### Statistical analysis

The data analysis for this manuscript was generated using SAS/STAT software, Version 9.1 of the SAS System for Windows (SAS Institute Inc., Cary, NC, USA). Chi-square analysis was performed using Fishers Exact option to determine if the proportion of positive SPT’s were different between groups. Antibody levels were compared using Analysis of Variance. All differences were considered significant at p < 0.05.

## Results

Of the 158 study participants recruited into the study, 101 had a confirmed clinical history of CRS and 57 control subjects had a clinical history of CIU but not CRS. In the CRS group, there were 59 females and 42 males. The age range was 19–70 and the mean age was 44.5. In the control group, there were 28 females and 29 males with an age range 33–61 and the mean age was 47. A total of 31 SPT antigen extracts were tested and 72 out of 99 (73%) CRS patients and 14 out of 44 (32%) CIU control patients reacted to at least one SPT extract (data not shown).

The SPT extracts most likely to elicit a positive SPT response in the CRS population were ragweed (40%), *A. alternata* (36%), cat (29%), dust mite (29%), and June grass (30%; Table 1). In the control population, ragweed (22%), cat (11%), June grass (21%), and dust mite (16%) extracts were the most common (Table 1). The SPT reactivity to the *A. alternata* extract was 3 fold higher in CRS compared to the control population. In the serological analysis, sIgE for the grass mix (21%) and tree mix (24%) was lower in CRS compared to control patients; however, there was a greater prevalence of fungal positive patients in CRS (21%) compared to the control population (16%) but this was not statistically significant (Table 2).

**Table 1:** Chi-squared analyses of skin prick test (SPT) reactivity among CRS patients (n = 99) and control patients (n = 44)

**SPT extract**	**CRS positive**^**†**^			**CRS negative**^**‡**^			**Odds ratio**	**Lower 95% CI**	**Upper 95% CI**	**p value**
	**n**	**n pos**	**%pos**	**n**	**n pos**	**%pos**				
*Perennial*										
** Cat**	**99**	**29**	**29.29**	**44**	**5**	**11.36**	**3.23**	**1.16**	**9.02**	**0.02**
Dust Mite	99	29	29.29	44	7	15.91	2.19	0.88	5.48	0.09
Dog	99	13	13.13	44	2	4.55	3.17	0.68	14.72	0.15
Cockroach	99	0	0	44	0	0	nc	nc	nc	nc
Mouse	99	0	0	44	0	0	nc	nc	nc	nc
*Seasonal*										
**Ragweed**	**99**	**40**	**40.40**	**44**	**10**	**22.73**	**2.31**	**1.02**	**5.19**	**0.05**
June	99	30	30.30	19	4	21.05	1.63	0.50	5.32	0.58
Black Birch	99	21	21.21	44	6	13.64	1.71	0.64	4.57	0.35
Timothy	99	19	19.19	43	3	6.98	3.16	0.88	11.34	0.08
Orchard	99	19	19.19	19	3	15.79	1.26	0.33	4.79	1
Sweet	99	19	19.19	19	2	10.53	2.02	0.43	9.49	0.52
Ash	99	16	16.6	20	3	15	1.09	0.29	4.17	1
Cocklebur	99	10	10.10	19	0	0	0.82	0.76	0.89	0.36
Maple	99	9	9.09	20	0	0	0.82	0.75	0.89	0.35
Willow	99	7	7.07	20	1	5.00	1.45	0.17	12.44	1.00
Oak	99	7	7.07	20	0	0	0.82	0.75	0.89	0.59
Poplar	99	5	5.05	20	1	5.00	1.01	0.11	9.15	1
Elm	99	4	4.04	20	0	0	0.83	0.76	0.89	1
Pine	99	2	2.02	20	1	5.00	0.39	0.03	4.54	0.43
*Fungal*										
***Alternaria alternata***	**99**	**36**	**36.36**	**43**	**5**	**11.63**	**4.34**	**1.57**	**12.02**	**0.003**
*Hormodendrum*	99	14	14.14	20	0	0	0.81	0.74	0.89	0.12
*Cephalosporium*	99	5	5.05	20	0	0	0.82	0.76	0.89	0.59
*Phoma*	99	5	5.05	20	0	0	0.82	0.76	0.89	0.59
*Aspergillus flavus*	99	4	4.04	20	0	0	0.83	0.76	0.89	1
*Fusarium*	99	4	4.04	20	0	0	0.83	0.76	0.89	1
*Penicillium*	99	3	3.03	20	0	0	0.82	0.76	0.89	1
*Cephalothecium*	99	2	2.02	20	0	0	0.82	0.76	0.90	1
*Helminthosporium*	99	2	2.02	20	0	0	0.83	0.76	0.90	1
*Rhizopus*	99	1	1.01	20	0	0	0.83	0.77	0.90	1
*Mucor*	99	1	1.01	20	0	0	0.83	0.77	0.9	1
*Monilia*	99	0	0	20	0	0	nc	nc	nc	nc

Two CRS patients (**†)** and 13 control subjects (**‡)** were not evaluated by SPT.

**Table 2:** Chi-squared analysis of human serum IgE reactivity to grass (gx1), mold (mx2), and tree (tx1) mix Phadia ImmunoCap antigens

IgE ImmunoCap Antigen*	**CRS positive**			**CRS negative**			**Odds ratio**	**Lower 95%CI**	**Upper 95% CI**	**p value**
	**n**	**n pos**	**%pos**	**n**	**n pos**	**%pos**				
Grass Mix (gx1)	101	21	20.79	57	13	22.81	0.89	0.41	1.95	0.84
Mold Mix (mx2)	101	21	20.79	57	9	15.79	1.40	0.59	3.31	0.53
Tree Mix (tx1)	101	24	23.76	57	15	26.32	0.87	0.41	1.84	0.84

*Serum IgE titers > 65 kUA/L were interpreted as positive to the tested Phadia ImmunoCap antigen.

In the chi-squared analysis, positive SPT responses to *A. alternata* were more prevalent in patients with CRS compared to controls (odds ratio (OR): 4.34, 95% confidence interval: 1.57, 12.02; Table 1). Sensitization to other perennial and seasonal SPT extracts including cat (OR: 3.23, 95% CI: 1.16, 9.02) and ragweed (OR: 2.31, 95% CI: 1.02, 5.19) was additionally identified to be more prevalent in the CRS group compared to the control group (Table 1). An elevated OR (1.4) was also observed following the analysis of IgE ImmunoCAP mold mix serological data; however, this was not statistically significant (Table 2). Differences between CRS and control patients were observed for other perennial and seasonal allergen sources including dog (OR: 3.17, 95% CI: 0.68, 14.72), dust mite (OR: 2.19, 95% CI: 0.88, 5.48), and timothy grass (OR: 3.16, 95% CI: 0.88, 11.34); however, these differences did not reach statistical significance and that may be due to the small sample size (Table 1). No other statistically significant differences were identified between the other allergen extracts tested in the SPT and serological analysis (Table 2).

In the ANOVA analysis of sIgE, the mean allergen specific units (kUA/L) measured for CRS and CIU control patients did not vary for grass (p = 0.67), mold (p = 0.34) or tree mix (p = 0.46) ImmunoCAP antigens (Table 3). Similarly, there were no statistically significant differences between mean sIgG titers (mg/mL) in CRS and control patients for *C. herbarum* (p = 0.84), *A. alternata* (p = 0.92), *S. atra* (p = 0.13), and *A. versicolor* (p = 0.22; Table 4).

**Table 3:** Analysis of variance of the mean allergen specific units (kUA/L) measured for CRS (n = 101) and control patients (n = 57)

**IgE ImmunoCap Antigen**	**CRS Positive**		**CRS Negative**		**F value**	**P value**
	**Mean allergen specific units (kUA/L)**	**Standard error**	**Mean allergen specific units (kUA/L)**	**Standard error**		
Grass Mix (GX1)	397.37	166.59	517.56	221.76	0.19	0.67
Mold Mix (MX2)	103.03	65.57	206.81	87.28	0.90	0.34
Tree Mix (TX1)	322.45	101.28	198.76	134.81	0.54	0.46

**Table 4:** Analysis of variance of the mean IgG titer (mg/mL) measured for CRS (n = 101) and control patients (n = 57)

**IgG ImmunoCap Antigen**	**CRS Positive**		**CRS Negative**		**F value**	**P value**
	**Mean IgG (mg/mL)**	**Standard error**	**Mean IgG (mg/mL)**	**Standard error**		
*Cladosporium herbarum* (GM2)	31.18	3.47	32.33	4.62	0.04	0.84
*Alternaria alternata* (GM6)	5.05	0.42	4.98	0.56	0.01	0.92
*Stachybotrys atra* (RGM24)	4.75	0.39	3.76	0.51	2.36	0.13
*Aspergillus versicolor* (RGM25)	23.76	2.66	18.54	3.41	1.53	0.22

## Discussion

In this study, 73% of CRS patients had a positive SPT to at least one of the tested allergen extracts. Sensitization to *A. alternata,* cat and ragweed were the most prevalent sensitizations in CRS patients compared to the control population. Sensitization to dust mite or timothy grass was also noted but these results were not statistically significant. These results highlight the potential importance of allergic sensitization to *A. alternata* and other perennial or seasonal allergen sources in CRS patients.

The prevalence of CRS patients having at least one positive SPT (73%) was comparable to results reported in a study that was located in close proximity (Cleveland, OH) to Toronto [9]. The identification of increased prevalence of fungal allergy in CRS was also identified in Cleveland as well as in southern regions of the United States [8,14,18] but not in Europe [19]. The serological results reported in the present study varied from previous studies reporting up to a 5 fold increase in IgG titers as well as increases in IgE^+^ cells (mast, plasma cells) in CRS patients [20,21]. The lack of concordance between SPT and serological studies in CRS patients may be due to increased sensitivity of SPT but also the variability of allergen extracts, as well as concurrent exposure of the subjects to antigens derived from a varying spectrum of perennial and seasonal allergen sources (tree, grass, insect, and fungal) [9]. Allergic sensitization to staphylococcal and streptococcal toxins has been found to be as high as 78% in CRS patients [22]. While these factors were not evaluated in the present study, they could also influence the pathology and prevalence of sensitization in CRS patients. Taking these limitations into consideration, the results of the present study suggest that evidence of allergic sensitization, in particular fungal hypersensitivity, could potentially be used as diagnostic criteria for CRS [8,18]. Currently, the Canadian clinical practice guidelines for CRS recognize allergy testing in cases of CRS but the guidelines do not recognize specific allergens [4].

In the present study, 36% of the CRS study participants were sensitized to *A. alternata*. These SPT results are comparable to other studies that have identified sensitization to *Alternaria* in CRS patients [20]. *A. alternata* has been previously implicated in cases of CRS in the United States [20] and it is among the most ubiquitous phyloplane fungal species in Toronto [23,24]. The presence of *A. alternata* conidia in the upper respiratory tract has been demonstrated in microscopic and immunologic studies of allergic mucin derived from CRS subjects [25-27]. *In vitro* studies have also shown that *A. alternata* extracts induce the recruitment of eosinophils, induce interferon-y, IL-5 and IL-13 in peripheral blood mononuclear cells [20,28]; and regulates the expression of surfactant protein D and LL-37 in fungal sensitized CRS patients [29,30]. It has been hypothesized that allergens released from *Alternaria* conidia and germinating conidia may have a functional role in CRS but this has remained controversial and is the subject of continued research [8,31].

Sensitization to cat and ragweed were additionally identified as prevalent sensitizations in the present study, and sensitization to these allergens has been previously reported in a CRS study in Washington D.C. [32]. Cat allergy is an important source of perennial allergen in inner city environments. Interestingly, Canadian aerobiological surveys have shown that the highest concentration of ragweed pollen occur in southern Ontario [33].

Our results deviate from previous studies that have identified dust mite to be an important perennial allergen associated with CRS [9,13,19,34]. Gutman and colleagues identified 73% of CRS patients in Cleveland to be sensitized to dust mite [9]. A study of 200 patients in San Francisco who had undergone functional endoscopic sinus surgery for chronic or recurrent rhinosinusitis showed that 22% were sensitized to house dust mite [13]. Freudenberger et al. [34] also identified 68% of CRS patients in New York to exhibit a positive immediate skin test reaction to dust mite extract. Although dust mite sensitization can be as high as 60% in Toronto [35], the prevalence of sensitization to dust mite only approached 30% in the present study and was not more prevalent in patients with CRS. To date, the role of seasonal and perennial allergy in CRS requires further evaluation; however, the results of this study suggest that in addition to *Alternaria*, cat and ragweed allergens may additionally contribute to swelling and blockage of sinus ostia leading to mucous buildup, secondary infection, and the development of CRS [4].

There were several limiting factors associated with the design of this study that should be considered when interpreting these results. The association of other seasonal and perennial allergens to CRS such as dust mite, dog and timothy grass may have been identified if a larger number of study participants and controls were recruited into the study. Selection and recruitment of patients included CRS patients with and without nasal polyps and allergic fungal sinusitis which may have resulted in the over estimation of allergic sensitization in this population of Canadian CRS patients [10,11,36]. However, our objective was to study the overall prevalence of allergy sensitization in CRS. Geographic variability in CRS and allergic sensitization are also variables that may have influenced the results of this study [5,6]. Surveys of CRS in Canada have shown a greater prevalence of CRS in eastern provinces, in particular Nova Scotia [5]. We hypothesize that the allergen burden in Nova Scotia may differ to the Toronto metropolitan environment due to a varying spectrum of tree, grass, weed, insect and mold allergens and as a result, the profile of allergic sensitization may vary substantially. Future studies should include larger patient numbers and utilize a wider panel of perennial and seasonal allergens that reflect the allergen burden of the local study environment.

## Conclusions

In conclusion, the results of this study demonstrate a general association between CRS and allergic sensitization. In the Toronto metropolitan area, *A. alternata* was prominent but cat and ragweed allergens were also associated with CRS. Taken together, these results suggest exposure to regionally specific allergens may drive a process resulting in CRS in some patients, perhaps with a genetic predisposition or epidermal barrier dysfunction. Future studies should be designed in other regions of Canada to identify and characterize the specific sensitization profiles of CRS patients.

## Competing interests

The authors declare no conflict of interest or competing interests.

## Authors’ contributions

BJG is a co-investigator with input on study design, interpreted statistical results, and prepared the manuscript. DHB is a co-investigator with input on study design and implementation, and assisted in the preparation and review of the manuscript. ZG enrolled patients, conducted regulatory work, and assisted in the collection of data, CSB conducted data analysis, reviewed patient charts, and reviewed the manuscript. RM recruited patients, conducted data analysis, reviewed patient charts, and reviewed the manuscript. TAB conducted the Phadia ImmunCAP serological analysis and reviewed the manuscript. MLK conducted the statistical analysis, interpreted and discussed results, and reviewed the manuscript. GLS is the principle investigator and contributed to the enrolment of patients, data capture and processing and has reviewed and commented on various drafts of the manuscript. All authors have read and approved the final manuscript.
